# Pharmacology, Toxicology, and Metabolism of Sennoside A, A Medicinal Plant-Derived Natural Compound

**DOI:** 10.3389/fphar.2021.714586

**Published:** 2021-10-26

**Authors:** Jiamei Le, Houlin Ji, Xiaoxiao Zhou, Xindong Wei, Yifan Chen, Yi Fu, Yujie Ma, Qiuqin Han, Yongning Sun, Yueqiu Gao, Hailong Wu

**Affiliations:** ^1^ Shanghai University of Medicine & Health Sciences Affiliated Zhoupu Hospital, Shanghai, China; ^2^ Shanghai Key Laboratory of Molecular Imaging, Collaborative Innovation Center for Biomedicine, Shanghai University of Medicine and Health Sciences, Shanghai, China; ^3^ Shanghai University of Traditional Chinese Medicine, Shanghai, China; ^4^ Nanjing University of Chinese Medicine Affiliated 81st Hospital, Nanjing, China; ^5^ Department of Cardiology, Shanghai Municipal Hospital of Traditional Chinese Medicine, Shanghai University of Traditional Chinese Medicine, Shanghai, China; ^6^ Department of Liver Diseases, Central Laboratory, Institute of Clinical Immunology, ShuGuang Hospital Affiliated to Shanghai University of Traditional Chinese Medicine, Shanghai, China; ^7^ Laboratory of Cellular Immunity, Shuguang Hospital, Shanghai University of Traditional Chinese Medicine, Shanghai, China

**Keywords:** sennoside A, dianthrone glycoside, pharmacology, toxicology, metabolism

## Abstract

Sennoside A (SA) is a natural dianthrone glycoside mainly from medicinal plants of Senna and Rhubarb, and used as a folk traditional irritant laxative and slimming health food. Accumulating evidences suggest that SA possesses numerous pharmacological properties, such as laxative, anti-obesity, hypoglycemic, hepatoprotective, anti-fibrotic, anti-inflammatory, anti-tumor, anti-bacterial, anti-fungal, anti-viral, and anti-neurodegenerative activities. These pharmacological effects lay the foundation for its potential application in treating a variety of diseases. However, numerous published studies suggest that a long-term use of SA in large doses may have some adverse effects, including the occurrence of melanosis coli and carcinogenesis of colon cancer, thereby limiting its clinical use. It remains to be established whether SA or its metabolites are responsible for the pharmacological and toxicity effects. In this review, the latest advances in the pharmacology, toxicology, and metabolism of SA were summarizedbased on its biological characteristics and mechanism.

## Introduction

Sennosides, a class of natural anthraquinone derivative and dimeric glycosides, are main bioactive components from medicinal plants used for traditional herbal laxatives, such as Senna *alexandrina* Mill. (Senna) and *Rheum Officinale* Baill (Rhubarb). Among them, sennoside A and B (SA, SB) are the main purgative components, which were first isolated and identified from the leaves of Senna and then attributed to the anthraquinone family by Stoll (Stoll et al., 1949). Later, other two pharmacologically active sennosides, including sennoside C and D (SC, SD), were isolated from the same plant senna (Lemli et al., 1981). In addition, SA, SB and SC have also been isolated from Rhubarb (Zwaving, 1965; Miyamoto et al., 1967; Oshio et al., 1974). All sennosides displayed almost comparable purgative effects in a bioassay with mice, probably due to the structure similarity (Oshio et al., 1978). However, SA and SB showed different biological activities. For example, SA but not SB could improve insulin resistance and suppress viral reverse transcriptase (Choi et al., 2006; Esposito et al., 2016). It was SB, but not SA, that inhibited cell proliferation in human osteosarcoma and the growth of *Entamoeba histolytica* trophozoite (Chen et al., 2009; Espinosa et al., 2020).

**FIGURE 1:**
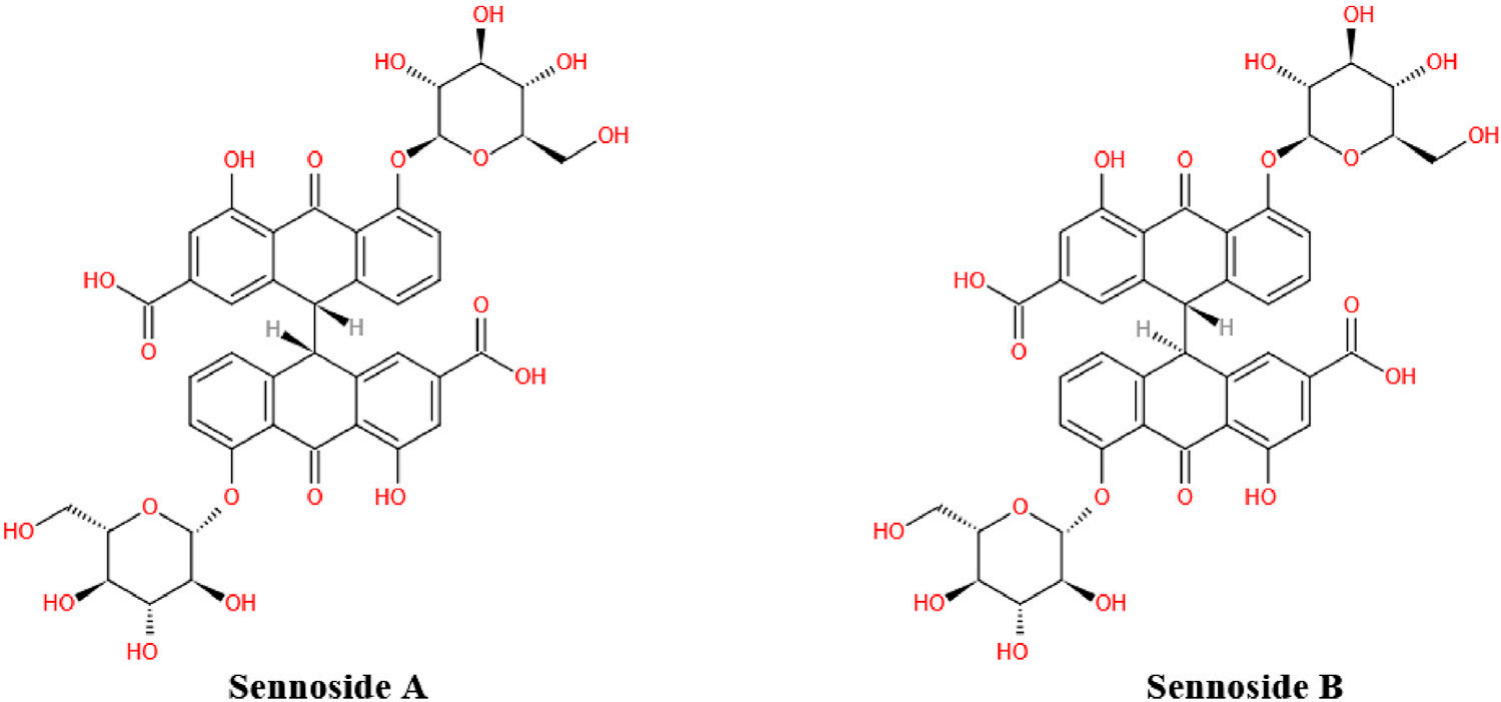
Chemical structure of sennoside A and B. Sennoside A (SA) and its stereoisomer sennoside B (SB) are natural anthraquinone derivative and dimeric glycosides.

**FIGURE 2:**
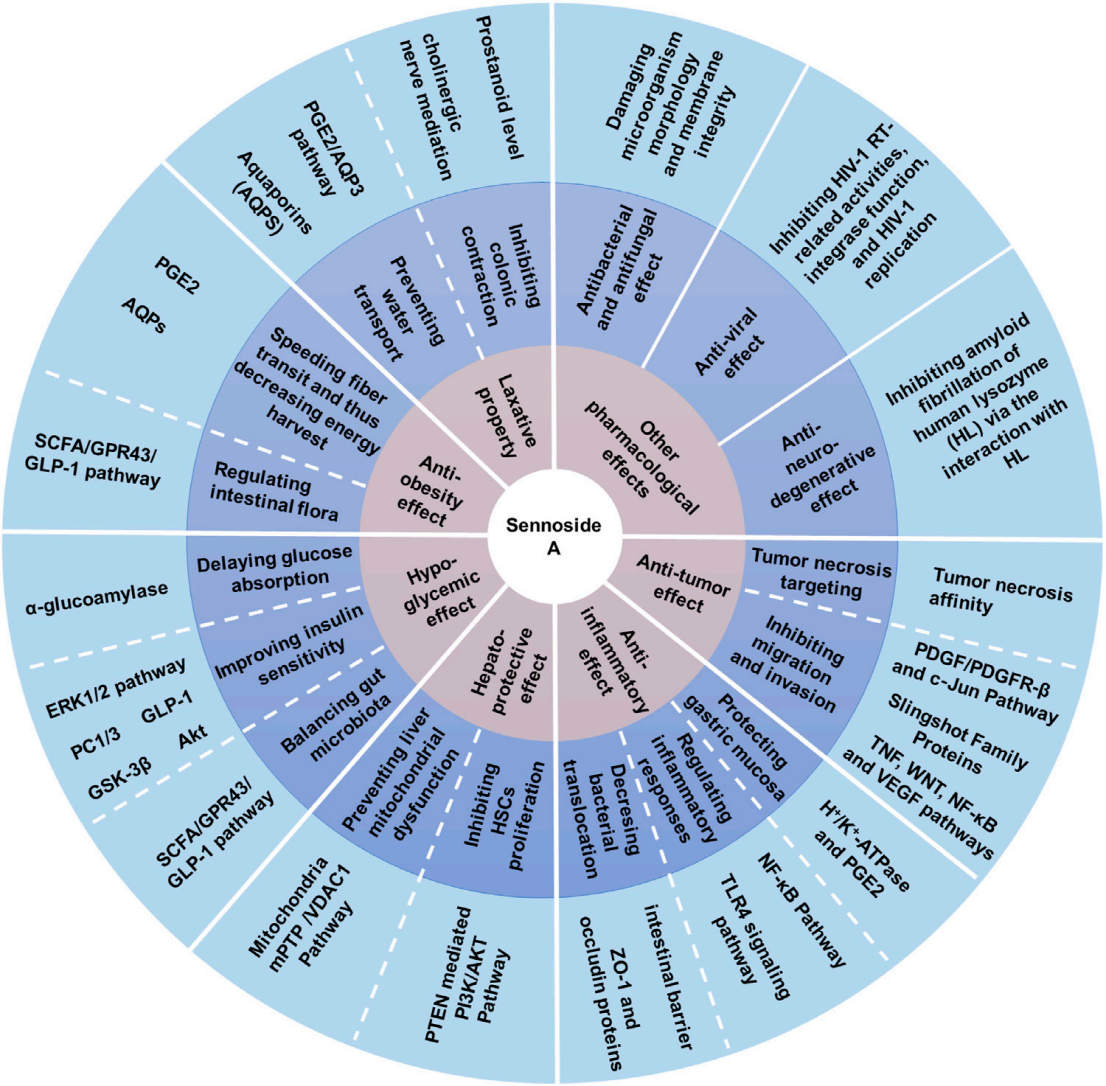
Pharmacological activities of sennoside A. SA has many potential pharmacological benefits, including laxative, anti-obesity, hypoglycemic, hepatoprotective, anti-inflammatory, anti-tumor, anti-bacterial, antifungal, anti-viral and anti-neurodegenerative effects.

As the most important family member of sennosides, SA is a type of irritant laxative, weight-loss herbal medicine or dietary supplement which has been routinely used for a long history in China and other Asian countries. The SA has some physicochemical properties of LogP: 1.88, molecular formula: C_42_H_38_O_20_, molecular weight: 862.7, melting point: 200–203°C, sparingly soluble in MeOH, insoluble in water and low bioavailability. Furthermore, SA can be slowly isomerized to its stereoisomer SB with the same molecular formulae and identical substituent (H) located in opposite directions, in NaHCO_3_ solution at 80°C (Sagara et al., 1987; Pubmed, 2004). The chemical structure of SA and SB are shown in Figure 1. In addition to the laxative effect (Kon et al., 2014; Cao et al., 2018), SA has been shown to possess other potential pharmacological and therapeutic applications, including anti-obesity (Greenway et al., 2006; Le et al., 2019; Wei et al., 2020), hypoglycemic (Choi et al., 2006; Le et al., 2019; Wei et al., 2020), hepatoprotective (Le et al., 2018; Zhu et al., 2020), anti-inflammatory (Chen et al., 1999; Hwang and Jeong, 2015; Kwon et al., 2016) and anti-cancer effects (Lee et al., 2017; Le et al., 2020) (Figure 2; Table 1 **).** Thus, SA has great potential to treat various illnesses, such as constipation, obesity, diabetes, fatty liver, many inflammatory and cancers. However, some other studies have suggested that high-dose and long-term use of SA may induce melanosis *coli* and subsequent colon cancer.

**TABLE 1:** The pharmacological activities of sennoside A.

Pharmacological effect	Cell lines/model	Activity/mechanism(s) of action	Application/dosage	Reference
The laxative property	DDY strain mice	Inhibiting contractions in the proximal colon	In vivo (30 mg/kg)	Kobayashi et al. (2007)
	HT-29cells, Raw264.7cells and Wistar rats	Increasing the secretion of PGE2 to decrease AQP3 expression	*In vitro* (200 μM) and vivo (50 mg/kg)	Kon et al. (2014)
	Sprague Dawley (SD) rats	Regulating AQPs expression	*In vivo* (50 mg/kg)	Cao et al. (2018)
Anti-obesity effect	HFD-induced obese mice, *db/db* mice C57BL/Ks mice	Reducing energy intake and regulating intestinal flora	*In vivo* (25, 30, 50 mg/kg)	Le et al., 2019; Wei et al., 2020
Hypoglycemic effect	STZ-induced diabetic mice	Inhibiting α-glucoamylase	*In vivo* (100 μM)	Choi et al. (2006)
	NCI-H716 cells and C57BL/6 mice	Activating ERK1/2 pathway to induce GLP-1 secretion	*In vitro* (1, 10, 100 μM) and vivo (15, 30, 45 mg/kg)	Ma et al. (2020b)
	HFD-induced obese mice	Stimulating SCFA/GPR43/GLP-1 pathway	*In vivo* (30 mg/kg)	Le et al. (2019)
	*db/db* mice and C57BL/Ks mice	Regulating gut bacteria and improving phosphorylated GSK-3β and Akt levels	*In vivo* (25, 50 mg/kg)	Wei et al. (2020)
Hepatoprotective effect	HFD-induced obese mice	Suppressing mitochondria mPTP/VDAC1 Pathway	*In vivo* (30 mg/kg)	Le et al. (2018)
	CCl4-induced liver fibrosis mice and HSC-T6 cell line	Inhibiting PTEN mediated PI3K/AKT Pathway	*In vitro* (10 μM) and vivo (15, 30, 60 mg/kg)	Zhu et al. (2020)
Anti-inflammatory effect	Acute pancreatitis rats	Inhibiting bacterial translocation	*In vivo* (0.1 g/ml sennoside solution)	Chen et al. (1999)
	HFD-induced obese mice, *db/db* mice and C57BL/Ks mice	Protecting intestinal barrier function	*In vivo* (25, 30, 50 mg/kg)	Ma et al., 2020a; Wei et al., 2020
	*db/db* mice and C57BL/Ks mice	Inhibiting TLR4 signaling pathway	*In vivo* (25, 50 mg/kg)	Wei et al. (2020)
	Acute reflux esophagitis SD rats	Suppressing NF-κB pathway	*In vivo* (RC-mixture containing 3.14% SA at a dosage of 100, 200, 400 mg/kg)	Kwon et al. (2016)
	HCl·EtOH-induced gastritis rats and indomethacin-induced gastric ulcers rats	Inhibiting H^+^/K^+^-ATPase and upregulating PGE2	*In vivo* (100 mg/kg)	Hwang and Jeong (2015)
Anti-tumor effect	Human pancreatic cancer cells (MIAPaCa-2 and Panc-1), human umbilical vein endothelial cells (HUVECs), liver metastasis animal model by spleen injection of Panc-1-Luc cells in BALB/c nu/nu nude mice	Inhibiting Slingshot (SSH) family proteins to increasing cofilin phosphorylation	*In vitro* (10 μM) and vivo (10 mg/kg)	Lee et al. (2017)
	HepG2 and SMMC-7721 cell line, orthotopic xenograft tumor model	Inhibiting Wnt, TNF, VEGF, and NF-κB signaling pathway	*In vitro* (100 μM) and vivo (10 mg/kg)	Le et al. (2020)
	KM mice and SD rats	Targeting tumor necrosis	*In vivo* (^131^I-SA, 1 mg/ml)	Yin et al. (2017)
Antibacterial and antifungal effect	Gram-positive bacteria, Gram-negative bacteria and fungi	Damaging microorganism morphology and membrane integrity	*In vitro* (100–400 g/ml)	Ram Avtar Sharma (2012)
Antiviral effect	Human T-lymphoblastoid Jurkat cell line, clone E6-1, and human embryonic kidney 293 T cell line	Inhibiting viral reverse transcriptase (RT)-related activities, integrase function, and the replication of virus	*In vitro* (20 μM)	Esposito et al. (2016)
Anti-neuro-degenerative effect	Human lysozyme (HL) as amyloid-forming model	Inhibiting amyloid fibrillation of HL	*In vitro* (0–20 μM)	Gao et al. (2021)

Given that SA is difficult to be absorbed by the gastrointestinal tract, it remains to be determined whether SA itself or its metabolites are responsible for its pharmacological and toxic effects. At present, the studies on SA mainly focused on the content determination methods and pharmacological actions, but the metabolism, and the pharmacological and toxic mechanism of SA are largely neglected. There is a lack of systematic evaluation about the pharmacology, toxicity, and metabolism of SA. Hence, we collected related studies on SA by using the keyword from globally recognized scientific databases, such as Web of Science, Springer, ScienceDirect, Elsevier. In this review, we described the up-to-date studies on the pharmacology, toxicity, and metabolism of SA, then discussed its therapeutic potential and safety in clinical applications.

## Pharmacology

### The Laxative Property

Chronic constipation is a prevalent gastrointestinal healthy issue, which can be easily treated by using over-the-counter laxatives (Jones et al., 2002). Sennosides, the most popular stimulant laxatives, can be transformed to an active metabolite, rhein anthrone, which leads to purgative action in the intestine. A study has shown that SA (30 mg/kg) inhibited contractions in the proximal colon, and in turn reduced the passage time of luminal contents and the absorption of water, hence resulting in accelerated transit of luminal contents in the distal colon. The mechanisms were associated with luminal prostanoid level and only partially with cholinergic nerve mediation (Kobayashi et al., 2007). In addition, SA (50 mg/kg) might decrease the colonic expression of aquaporins (AQPs), consequently inhibiting the lumen-to-blood water transportation and leading to defecation. The main mechanism was that the SA-derived metabolite, rhein anthrone, could activate macrophages in the colon to secrete prostaglandin E2 (PGE2), which in turn acted as a paracrine factor to downregulate the levels of AQP3 in the epithelia of colon mucosa (Kon et al., 2014).

Aquaporins (AQPs), a family of membrane proteins, can form water channels on the cell membrane and play a key role in regulating water transport within and between cells (Agre, 2006). Up to now, at least 13 AQPs have been identified in the digestive tract of mammals, and possessed various physiological functions including secretion of gastric juice, transportation of water, and absorption or secretion of water and small solutes through the epithelia (Zhu et al., 2016). *Cao et al.* reported that, as the well-known effective laxatives, the senna and its main components sennosides could target multiple AQPs to achieve their treatment effects on constipation. These results showed that the senna extracts (content of SA: 41.3 mg/kg), sennosides (content of SA: 25.52 mg/kg), and a monomer active component SA (50 mg/kg) could prevent water transition from colon lumen to epithelia by regulating multiple colonic AQPs expressions, subsequently leading to anti-constipation effect (Cao et al., 2018). However, the observed results demonstrated that the treatment of senna extracts might cause toxicity on the kidney and liver, while SA mediated AQP9 upregulation could improve senna extract-mediated liver injury (Cao et al., 2018).

### Anti-Obesity Effect

Obesity is a major public health issue worldwide influenced by excessive calorie intake and lack of physical exercise. Obesity can cause multiple metabolic disorders, such as hyperglycemia, insulin resistance, and chronic systemic inflammation, which can be complicated by a series of metabolic syndrome diseases, such as non-insulin-dependent diabetes mellitus, arthritis and cancer (Conway and Rene, 2004). Several reports found that SA might decrease energy intake from intestine by accelerating transit of luminal contents in the distal colon (Rumsey et al., 1993; Kobayashi et al., 2007). Moreover, SA was reported to be a common ingredient in both herbal medicines and dietary supplements for weight-loss (Greenway et al., 2006; Kim et al., 2014).

Fecal microbiota transplantation (FMT) can transfer the obesity phenotype from obese humans to sterile mice, suggesting that gut microbiota plays a decisive role in the occurrence or development of obesity and other metabolic disorders (Ridaura et al., 2013). Previous studies also verified that SA (25, 30, or 50 mg/kg) could alleviate obesity traits possibly by regulating intestinal flora (Le et al., 2019; Wei et al., 2020). The current study also found that SA (30 mg/kg) could restore the secretion of glucagon-like peptide-1 (GLP-1), accompanied by decreased body weight (Le et al., 2019). This finding was agreed with a previous study by Otten et al. showing that weight-loss was in parallel with an increase in postprandial GLP-1 and a further rise of GLP-1 occurred during weight maintenance (Otten et al., 2019).

### Hypoglycemic Effect

As a type of highly heterogeneous metabolic disorder, type 2 diabetes (T2DM) is characterized with the impairment of insulin secretion and the development of insulin resistance (Kahn, 2003). Extracts from Rhubarb (chrysophanol) had been shown to improve multiple metabolic disorders, such as hypercholesterolemia, diabetic nephropathy, and platelet aggregation (Su et al., 2020). Meanwhile, SA, another active ingredient of Rhubarb, was found to exert a positive effect in the treatment of T2DM (Choi et al., 2006; Le et al., 2019; Wei et al., 2020).

#### α-Glucoamylase

Inhibition of α-glucoamylase is greatly attributed to the therapeutic effects of hypoglycemic agents in decreasing post-prandial hyperglycemia (Choi et al., 2006). Choi et al. have found that the extracts from Rhubarb might improve insulin sensitivity and delay carbohydrate digestion by suppressing the activity of α-glycosylase in diabetic mice induced by streptozotocin (STZ), thereby improving glucose tolerance. Among the Rhubarb extracts, SA (100 μM) acted as a strong inhibitor of α-glucoamylase similar to acarbose *in vitro* (Choi et al., 2006).

#### The Extracellular Signal Regulated Kinase Signaling Pathway

The extracellular signal regulated kinase (ERK1/2) pathway is involved in a variety of physiological processes involved in cell proliferation, development and differentiation by regulating various downstream targets (Roskoski, 2012). Blockage of ERK1/2 signaling pathway could reduce GLP-1 secretion in NCIH716 cells under insulin stimulation (Lim et al., 2009). Consistently, Ma et al. demonstrated that SA (45 mg/kg *in vivo* and 100 μM *in vitro*) stimulated the secretion of GLP-1 in L cells by activating ERK1/2 pathway and improving the expression of PC1/3 protein, which might be the main mechanism of improving insulin sensitivity. However, the activation mechanism of ERK1/2 and its effect on PC1/3 have not been elaborated in detail (Ma et al., 2020b).

#### Glucagon-Like Peptide 1

Glucagon-like peptide 1 (GLP-1), an type of intestinal hormone in L cells, is encoded by proglucagon gene and produced in a nutrient-dependent manner (Drucker, 2006). The impaired GLP-1 response contributes to the pathogenesis of T2DM (Vilsbøll et al., 2001). As previously indicated, structural damage in the colon mucosa has resulted in a reduction on the secretion of GLP-1 in a high-fat diet (HFD) induced obese (DIO) mouse model (Le et al., 2019). The long-term high-fat diet could induce adenosine triphosphate (ATP) production and mitochondrial damage. This also leads to the dysfunction of microbial energy metabolism in the large intestine, then increasing the risk of insulin resistance and T2DM (Le et al., 2019). As one of the short-chain fatty acids (SCFAs), butyric acid restored by SA could lead to decreased reactive oxygen species (ROS) during the production process of ATP in mitochondria, effectively protecting the intestinal epithelial cells of DIO mice (Le et al., 2019). It has been reported that SCFAs also promoted the secretion of GLP-1 in intestinal epithelial cells by stimulating G protein-coupled receptors (GPR43 or GPR41) (Nøhr et al., 2013). Based on our previous research, SA (30 mg/kg) could stimulate the secretion of GLP-1 by protecting the balance of gut microbiota and improving SCFAs levels in DIO mice, which ultimately lead to improved insulin sensitivity (Le et al., 2019).

#### Gut Microbiota

Gut microbiota is an important metabolic “organ” of human body and plays a role in fermenting carbohydrates, regulating intestinal movement, and synthesizing trace elements. The disturbances of gut microbiota could cause adipose tissue inflammation and fat metabolism dysregulation, which in turn lead to insulin resistance and diabetes (Sonnenburg and Bäckhed, 2016). The previous study reported that SA (30 mg/kg) improved insulin sensitivity by regulating intestinal flora (Le et al., 2019). In addition, SA (25, 50 mg/kg) has been reported to regulate the levels of blood glucose and attenuate the traits of T2DM and obesity in *db/db* mice by modulating the composition of gut microbiota. The levels of *Akkermansia*, *Odoribacter*, *Mucispirillum*, *Turicibacter* and SMB53 (good bugs) were significantly increased in the SA treatment group, while the levels of *Ruminococcus*, *Oscillospira* and AF12 (bad bugs) were significantly decreased. Unfortunately, the regulation of SA in specific single microbe at the species level has not been identified. The fecal microbiota transplantation (FMT) from SA-treated mice and the direct intragastric administration of SA could both correct the metabolic disorder and exert a significant hypoglycemic effect (Wei et al., 2020). Meanwhile, SA may improve insulin sensitivity by increasing the phosphorylation levels of glycogen synthase kinase 3 beta (GSK-3β) and protein kinase B (Akt) (Wei et al., 2020).

### Hepatoprotective Effect

Excessive hepatic fat accumulation is the main cause of non-alcoholic fatty liver disease (NAFLD), which gradually progresses to nonalcoholic steatohepatitis (NASH), liver fibrosis, and eventually results in liver cirrhosis and hepatocellular carcinoma (HCC) (Roeb, 2021). The SA showed mitochondrial protective effect and inhibited hepatic steatosis (Le et al., 2018). Concurrently, SA could also inhibit the proliferation of hepatic stellate cells (HSCs) and suppress the progression of liver fibrosis (Zhu et al., 2020).

#### Mitochondrial Related Pathway

Mitochondrial dysfunction is one of the earliest and the most detrimental events in the progression of fat diseases, because it can cause impaired cellular lipid homeostasis and excessive production of ROS in the liver (Einer et al., 2018). Under physiological conditions, mitochondrial Ca^2+^ influx activates the Krebs cycle to generate energy, and simultaneously produce a small amount of ROS, which is necessary for maintaining cell activities. The mitochondrial permeability transition pore (mPTP) can control the homeostatic mitochondrial Ca^2+^ efflux, thus regulating mitochondrial Ca^2+^ concentration and the activation of Ca^2+^-dependent dehydrogenases. However, constant mPTP opening will cause excessive release of mitochondrial matrix calcium and loss of mitochondrial membrane potential, resulting in mitochondrial swelling, outer mitochondrial membrane rupture and ATP production failure (Bravo-Sagua et al., 2017).

Voltage-dependent anion channel-1(VDAC1), a proposed component of the mPTP, plays important roles in regulating cellular apoptosis and energy metabolism (Shoshan-Barmatz et al., 2010). Knockdown of *VDAC1* significantly inhibited the human T-REx-293 cells growth and reduced ATP production (Abu-Hamad et al., 2006). Our research suggested that SA (30 mg/kg) protected the liver mitochondrial structure and function by targeting the mitochondrial/VDAC1 Pathway, suggesting that VDAC1 inhibition might contribute to SA-mediated mitochondrial protection in mice with HFD-induced hepatic steatosis (Le et al., 2018). The specific mechanism might be related to the inhibition of mitochondrial respiratory chain complex I via suppressing the expression of VDAC1 so as to prevent “ energy surplus” by SA, which ultimately decreased the liver weight and improved hepatic steatosis (Le et al., 2018).

#### The Phosphatase and Tensin Homolog Mediated Pathway

The persistent liver injury and wound-healing form a vicious circle leading to liver fibrosis (Zhou et al., 2014; Baglieri et al., 2019). It has been well recognized that HSCs exerted a vital effect in the pathogenesis of liver fibrosis. Therefore, inhibition of the activation, proliferation and function of HSCs would become a promising therapeutic method for liver fibrosis (Omar et al., 2016). The phosphatidylinositol-3-kinase (PI3K)/Akt signaling plays an important role in HSC proliferation and activation. The activation of PI3K/Akt signaling could promote proliferation and inhibit apoptosis in HSCs, which in turn contributes to the pathogenesis of liver fibrosis (Zhang et al., 2019). The phosphatase and tensin homolog (PTEN) is a major negative regulator of the PI3K/Akt signaling pathway. A previous study demonstrated that DNA methyltransferase 1 (DNMT1) could cause hypermethylation at the promoter region of PTEN, resulting in decreased PTEN expression and subsequent activation of the PI3K/Akt signaling in activated HSCs (Bian et al., 2012). Interestingly, by using surface plasmonic resonance assay, Zhu et al. showed that SA (30 mg/kg *in vivo* and 10 μM *in vitro*) could directly interact with DNMT1 and inhibit DNMT1 in HSCs, resulting in restored PTEN expression and decreased PI3K/Akt activation. Therefore, SA may serve as a promising natural supplement for the treatment of liver fibrosis (Zhu et al., 2020).

### Anti-Inflammatory Effect

Inflammation is the cornerstone of numerous physiological and pathological processes, and involved in various diseases, including obesity, T2DM, cardiovascular diseases and cancers (Medzhitov, 2008). The low-grade inflammation has been detected in the many metabolic diseases, and the improvement of adipose tissue inflammation could inhibit insulin resistance and weight gain in obesity (Xu et al., 2003; Strissel et al., 2007; Gregor and Hotamisligil, 2011). The anti-inflammatory effect of many extracts from medicinal plants has attracted the attention of researchers and applied in clinic widely. Some previous studies have demonstrated the anti-inflammatory effects of SA in the pathogenesis of pancreatitis, gastritis, reflux esophagitis and chronic low-grade inflammation (Chen et al., 1999; Hwang and Jeong, 2015; Kwon et al., 2016; Wei et al., 2020).

#### Bacterial Translocation

The intestinal track serves as a reservoir of endotoxins and bacteria. Under certain pathological conditions, such as liver cirrhosis, severe pancreatitis, paralytic ileus, bacteria or their pathogenic products can be transported into circulation, leading to systemic complications (Wiest et al., 2014; Liu et al., 2019; Tahara et al., 2019). In an acute pancreatitis rat model induced by intraductal infusion of sodium deoxycholate, sennosides (0.1 g/ml sennoside solution) treatment could restore peristalsis, increase mucus secretion, and reduce mortality, which was possibly due to decreased the circulation transport of endotoxin and bacteria (Chen et al., 1999).

#### Function of Intestinal Barrier

The intestinal barrier consists of monolayer intestinal epithelial cell and paracellular space, which blocks the passage of potentially harmful substances (Salvo Romero et al., 2015). Once the impairment of intestinal barrier occurs, harmful substances, such as endotoxin and microbiota, may enter the circulation, resulting in chronic low-grade inflammation (Tilg et al., 2020). Some recent studies have suggested that SA may protect the function of intestinal barrier (Ma et al., 2020a; Wei et al., 2020). Ma et al. reported that SA (30 mg/kg) could protect colon enterocytes from ROS toxicity and restore colonic barrier function, thereby improving insulin sensitivity induced low-grade chronic inflammation (Ma et al., 2020a). Similarly, Wei et al. suggested that SA (25, 50 mg/kg) decreased tissue inflammation by upregulating ZO-1 and occludin protein levels and protecting intestinal barrier function (Wei et al., 2020).

#### Toll-like Receptor 4 Signaling Pathway

Toll-like receptors (TLRs) are a class of receptors called pattern recognition receptor (PRRs), which recognize pathogen molecules to trigger the inflammatory responses and the activation of innate and adaptive immune system (Chen et al., 2018). Among them, the TLR4 signaling exerts a vital effect in inflammation in response to bacterial infection. Wei et al. found that SA (25, 50 mg/kg) reduced the expression of proinflammatory factors such as IL-6, MCP-1 and TNF-α, by regulating TLR4 signaling pathway (Wei et al., 2020). The stated result was consistent with a previous study showing that an extensive intracellular signaling cascade was initiated by the activation of TLR4, which activated the downstream targets in inflammatory signaling pathways and their interaction with insulin signaling pathways (Kopp et al., 2010).

#### Nuclear Factor Kappa B Pathway

Despite the continuous development of modern medicine, reflux esophagitis is still a worldwide problem, which seriously affects the quality of life of patients (Altomare et al., 2013). Nuclear factor kappa B (NF-κB), a transcription factor associated with immune responses, plays an key role in some inflammatory diseases, such as inflammatory bowel disease, autoimmunity, and rheumatoid arthritis (Mitchell and Carmody, 2018). Rhei Rhizoma and Coptidis Rhizoma Mixture (RC-mix), containing 3.14% SA at a dosage of 100, 200, 400 mg/kg, exerted significant antioxidant and anti-inflammatory activities in acute reflux esophagitis model. The RC-mix (containing SA) also protected against esophageal mucosal damage via the suppression of NF-κB by inhibiting IκBα phosphorylation, which in turn reduced the proinflammatory cytokines and mediators release (Kwon et al., 2016).

#### H^+^/K^+^-ATPase and PGE2

Chronic autoimmune gastritis (AIG) is characterized by an increase in inflammatory infiltration in the gastric mucosa, and the destruction or loss of parietal cells. The H^+^/K^+^-ATPase on parietal cells has been identified as the major humoral autoantigen in both AIG patients and experimental animal models (D'Elios et al., 2001). Hydrogen pump inhibitors act on gastric mucosal parietal cells, reducing the activity of H^+^/K^+^-ATPase and inhibiting the secretion of gastric acid. The proper inhibition of gastric secretion could promote the healing of gastric lesion and protect integrality of gastric mucosa (Engevik et al., 2020). In addition, nonsteroidal anti-inflammatory drugs (NSAIDs) such as ibuprofen, naproxen and aspirin, showed to enhance damage of gastric epithelial cell by inhibiting the activity of cyclo-oxygenase (COX) and the production of its downstream target, prostaglandin (PG) (Kimmey, 1992). The PG is highly expressed in gastric mucosa, which plays a key role in maintaining gastric mucosa blood flow, repairing mucus secretion, and promoting mucosal injury healing (Wallace, 2008; Takeuchi, 2014). In animal models, both SA (100 mg/kg) and SB (100 mg/kg) have shown to improve gastritis induced by HCl EtOH and gastric ulcer induced by indomethacin via increasing PGE2 expression and inhibiting H^+^/K^+^-ATPase (Hwang and Jeong, 2015).

### Anti-Tumor Effect

Tumor growth, proliferation, invasion, and metastasis are regulated by many factors. With the increasing incidence of cancer worldwide, it is necessary to develop potential drugs that can effectively treat and prevent the occurrence and progression of tumors. In recent years, it has been found that sennoside could function as an anti-tumor agent (Chen et al., 2009; Lee et al., 2017; Xu et al., 2018).

#### Slingshot Family Proteins

Cofilin is a well-known actin-modulating protein, and the activation of cofilin is required for cell migration. The activation or inactivation of cofilin are tightly regulated by dephosphorylation and phosphorylation at residue Ser-3 respectively. The phospho-cofilin (Ser-3) does not bind to actin, whereas the dephospho-cofilin binds to actin and promotes actin depolymerization, and in turn cell migration (Ghosh et al., 2004). The phosphatase slingshot homologs (SSHs) are responsible for transforming inactive cofilin into active one by dephosphorylation of phospho-cofilin (Ser-3) (Carlier et al., 1997). A recent study has identified SA as a novel and effective inhibitor of SSHs. The treatment of SA (10 mg/kg *in vivo* and 10 μM *in vitro*) could block dephosphorylation of phosphor-cofilin, impair actin dynamics and inhibit motility and invasiveness in pancreatic cancer cells (Lee et al., 2017).

#### Tumor Growth Related Signaling Pathways

Platelet-derived growth factors (PDGFs) and their receptors (PDGFR) participate in angiogenesis, which is one of hallmarks of cancer (Board and Jayson, 2005). As an inhibitor of PDGFR-β, SB (0.3–5 μM, the diastereomer of SA) has shown to inhibit PDGF-induced cell proliferation, and suppress the expression of downstream target genes of PDGF signaling, such as AKT, STAT-5 and ERK1/2. Interestingly, compared to SB, SA was far less potent in inhibiting the phosphorylation of PDGFR-β induced by PDGF-BB (Chen et al., 2009). The activator protein-1 transcription complex (AP-1) transcription factor subunit (c-Jun) regulates the progression of cell cycle through transactivation of cyclin D1, an important cell cycle regulator with overexpression in several cancers (Bakiri et al., 2000). A recent study demonstrated that SB could inhibit human osteosarcoma cell growth and metastasis, and induce G1 cell cycle arrest by inhibiting c-Jun expression and subsequent cyclin D1 expression (Xu et al., 2018).

#### Tumor Metastasis Related Signaling Pathways

Recently, the results demonstrated the inhibitory function of SA (10 mg/kg *in vivo* and 100 μM *in vitro*) on HCC cells growth, migration and invasion. The RNA sequencing (RNA-seq) data further revealed 9 metastasis-related differentially expressed genes (DEGs) which might be subjected to SA regulation and contributed to SA-mediated HCC inhibition. Among these 9 DEGs associated with tumor metastasis, keratin 7 (KRT7) and keratin 81 (KRT81) were confirmed to be related to HCC metastasis (Le et al., 2020). The results were consistent with previous studies that suggested that KRT7 and KRT 81 were expressed at increased levels in several types of cancers and contributed to cancer metastasis (Nanashima et al., 2017; Chen et al., 2020). Besides, several main KEGG enrichment pathways were shown to participate in the SA-mediated inhibitory effect on the HCC metastasis in our study, including tumor necrosis factor (TNF), vascular endothelial factor (VEGF), WNT and NF-κB signaling pathways (Le et al., 2020), which were reported to be related to tumor metastasis (Lebrec et al., 2015; Wang et al., 2018; Zhang et al., 2018; Wang et al., 2019).

#### Targeting of Tumor Necrosis

The targeted therapy for tumor necrosis has developed rapidly in recent years due to inducing regression or destruction of residual tumors. The SA (0.2 mg/ml) has been reported to possess strong avidity for necrotic tissue, which makes it very potent to serve as a necrosis-avid contrast compound for myocardial infarction imaging (Wang et al., 2013). Similarly, iodine-131-labeled SA (^131^I-SA, 1 mg/ml) and iodine-131-labeled sennidin A (^131^I-sennidin A, the aglycone of SA, 2 mg/ml) had potential antineoplastic activity, which might combine necrosis inducing drugs to exert synergistic tumoricidal action on solid malignancies (Ji et al., 2014; Yin et al., 2017).

### Other Pharmacological Effects

Sennoside A has been widely used as laxative drugs and health care products for weight loss. In recent years, it has been found that SA also has other pharmacological effects, including antibacterial, antiviral, anti-amoebic and anti-neurodegenerative effects (Raycroft et al., 2012; Esposito et al., 2016; Espinosa et al., 2020; Gao et al., 2021).

#### Antibacterial and Antifungal Effects

It has been reported that sennosides including SA (100–400 g/ml) had an inhibitory effect on a wide range of bacteria and fungi, such as *Salmonella typhi*, *Staphylococcus aureus (S. aureus)*, *Pseudomonas aeruginosa*, *Escherichia coli (E. coli)*, *Streptococcus pneumoniae*, *Bacillus subtilis*, *Rhizoctonia bataticola*, *Fusarium moniliforme*, *Aspergillus niger*, *Aspergillus flavus*, *Candida albicans* (Ram Avtar Sharma, 2012). In addition, SA-capped silver nanoparticles (Ag/SA NPs), synthesized with SA as reducing and capping agent at room temperature, significantly inhibited two bacteria strains (*S. aureus* and *E. coli*) and two yeast strains (*Candida albicans* and *Candida parapsilosis*) (Al-Ghamdi et al., 2020). Similarly, Ontong et al. showed that AgNPs, made the extracts of senna leaf as reducing and capping agents, exhibit strong inhibitory effects against various Gram-positive or -negative bacteria and fungi, by impairing the microorganism morphology and membrane integrity (Ontong et al., 2019).

#### Antiviral Effect

The reverse transcriptase (RT) is a type of critical enzyme in retrovirus and responsible for their replication by reverse transcription of RNA genome into viral DNA double-strand. Therefore, the RT is an attractive target for antiviral treatment (Esposito et al., 2012). Esposito *et al.* successfully identified SA (20 μM) as the chemical component in Rhubarb that could inhibit the activity of HIV-1 RT-related DNA polymerase activity and Ribonuclease H (RNase H) activity, resulting in reduced HIV-1 replication (Esposito et al., 2016).

#### Anti-Amebic Effect

Anti-amebic drugs currently used in clinic (such as paromomycin, iodoquinol, diloxanide furoate and nitroimidazoles) might generate resistance (Wassmann et al., 1999), trigger DNA mutations and thereby lead to cancer in laboratory animals (Bendesky et al., 2002). Furthermore, they could cause serious side effects on patients, such as vomiting, nausea, vertigo, headaches, dizziness, neuronal damage (Ali and Nozaki, 2007). Espinos et al. demonstrated that SB (the diastereomer of SA) might serve as an alternative of anti-amebic drugs. SB (60, 120 μM) inhibited the growth of *E. histolytica* trophozoite by producing toxic free oxygen metabolites in the ameba to either alter the activity of DNA replication/repair enzymes or directly damage the structure and function of DNA. The inhibition efficacy was comparable to that of metronidazole, a commercial anti-amebic drug (Espinosa et al., 2020).

#### Anti-Neurodegenerative Effect

Under the influence of aging and pathological factors, the formation of amyloid-like aggregates from misfolded proteins can frequently lead to some serious neurodegenerative diseases, such as Parkinson’s disease, Alzheimer’s disease, Huntington disease and Creutzfeldt-Jakob disease (Chiti and Dobson, 2017). Thus, the inhibition of amyloid fibrillation is the key to the treatment of many neurodegenerative diseases. In a previous study by using human lysozyme (HL) as amyloid-forming model, SA and SC (0–20 μM) showed to interact with HL via its binding pocket by multiple non-covalent bonds, such as van-der-Waals forces, hydrogen bonds and hydrophobic bonds. Therefore, SA and SC could inhibit the amyloid fibrillation of HL by stabilizing HL (Gao et al., 2021).

## Toxicology

The safety of sennosides containing 87% SA + SB and 5% SA + SC + SD had been evaluated in a wide range of toxicity studies by Mengs (Mengs, 1988). Mutagenicity assays showed that the sennosides (max. 5000 μg/ml, 87% SA + SB) had no genetic toxicity to microorganism and mammalian cells *in vitro* (Mengs, 1988). The reproduction toxicity studies showed that oral sennosides (2–100 mg/kg) had no embryo lethal or teratogenic toxicity on rabbits and rats. Oral sennosides (2–20 mg/kg, 87% SA + SB) had no adverse effects on the postnatal development of offspring, the rearing behavior of female animals and fertility toxicity (Mengs, 1988). Sennosides also showed no obvious specific toxicity in subacute and chronic toxicity studies (Mengs, 1988). However, the overdose of sennosides could induce slight gastrointestinal toxicity in acute toxicity studies. The death caused by overdose might be due to the loss of water and electrolytes induced by diarrhea. The calculated LD_50_ values were approximately 5,000 mg/kg in both rats and mice, which were consistent with the study of Marvola et al. showing that the LD_50_ was greater than 5,000 mg/kg in mice (Mengs, 1988; Marvola et al., 2011).

Similar to Mengs’s findings, another study by Tikkanen et al. proved that SA had no mutagenicity with or without metabolic activation in the *Salmonella typhimurium* co-culture test (Tikkanen et al., 1983). In this year, one clinical case reported that a 33 year-old woman have developed digital clubbing with elevated PGE2 after taking sennoside A + B calcium for constipation. Interestingly, the patient’s urinary PGE2 level returned to normal and clubbing symptoms improved after discontinuation of the drug, which suggested that clubbed digits was probably caused by sennoside A + B calcium (Kawamoto et al., 2021). Another two clinical case reported that sennosides (SA and SB) might induce hemorrhagic colitis with spontaneously regression after discontinuation (Villand, 1985; Ozdil et al., 2010). Nevertheless, Vilanova-Sanchez et al. reviewed information regarding perineal blisters associated with Senna as well as other secondary effects of long-term use of sena laxatives in children. They concluded that it has a low risk of adverse side effects and is safe to use under medical supervision, with even mild adverse effects self-limited with withdrawal of Senna (Vilanova-Sanchez et al., 2018).

The imbalance of cell proliferation and apoptosis is related to the process of carcinogenesis. Persistent overexpression of p53, a key protein to induce cell cycle arrest and apoptosis, could lead to the increased risk of colorectal cancer (Kastan et al., 1991; Chen et al., 1995; Nakayama and Oshima, 2019). van Gorkom et al. discovered that the apoptosis induced by short-term use of sennosides (2 mg/kg SA and SB) might be mediated through the p53-p21/WAF pathway. Although the colonic self-repair damage induced by a single high-dose sennoside laxative, long-term use of sennoside laxative would lead to constant overexpression of p53 and p21, which might lead to increased risk of colorectal cancer (van Gorkom et al., 2001).

The first step leading toward carcinogenesis was thought to be abnormal proliferation activity of epithelial cells, which has been related to increased risk of colon cancer (Preston-Martin et al., 1990). Toyoda *et al.* reported that the regenerative processes occurred after inflammatory or cytotoxic changes in response to laxatives (containing SA) stimulation would be responsible for inducing cell proliferation (Toyoda et al., 1994). Therefore, the abuse of laxatives containing anthraquinone glycosides could increase the risk of colon cancer (Kleibeuker et al., 1995). Aberrant crypt foci (ACF) had been recognized as the potential preneoplastic lesions in evaluating the induction and modulation of colon carcinogenesis (Magnuson et al., 1993). Senna glycosides (SA and SB) have been reported to promote the cell proliferation of ACF induced by 1,2-dimehtylhydrazine, and increased the average crypt number of each lesion (Mereto et al., 1996).

Mounting studies have shown that long-term use of anthraquinone laxatives to treat constipation could cause melanosis *coli* (MC), characterized by the presence of nonspecific brown pigments in the macrophages of colonic lamina propria and submucosa, which has been related to an increased risk of colorectal cancer (Biernacka-Wawrzonek et al., 2017; Cheng et al., 2020). Walker et al. suggested that synthetic anthraquinone danthron induced the epithelial cells apoptosis to produce apoptotic bodies, which were phagocytosed and degraded into lipofuscin by macrophages, participating in the formation of MC (Walker et al., 1988). *Cheng et al* reported serious damage in colon, including goblet cell reduction and inflammatory cell infiltration in crypt, after oral administration of rhubarb anthraquinone (the content of SA is 4.56%) for 90 days. They further demonstrated that SA metabolite rhein but not SA could induce autophagy and apoptosis in normal colon cells, which might lead to the formation of MC (Cheng et al., 2020).

**FIGURE 3:**
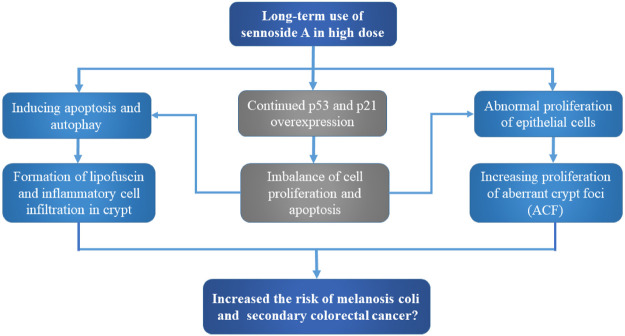
Potential carcinogenic toxicity and mechanism of sennoside A. Although still uncertain, SA may cause melanosis coli and subsequent colon cancer. The potential toxic mechanisms involve the aberrant cell proliferation and apoptosis, as well as inflammatory cell infiltration and aberrant crypt foci.

Generally, there is no solid evidence so far to support the link between exposure of senna, senna extracts or sennosides and tumor formation in rodent models. For instance, no intestinal neoplastic changes had been observed in both wild-type and P53^+/−^ mice with administration of senna (0.7% SA) in diets (Surh et al., 2013). In another 2 year study, no intestinal lesions were induced in Sprague-Dawley rats by the administration of a senna extract (35–42% sennosides) in drinking water (Lydén-Sokolowski et al., 1993). According to Borrelli et al., no ACF or tumors were observed in male Wistar rats after oral administration of senna extracts (30 or 60 mg/kg) 6 times per week with for 110 weeks (Borrelli et al., 2005). So, it remains unconfirmed that the long-term use of SA in high doses induces intestinal hyperplasia and increases potential risk to cause MC and subsequent colon cancer (Figure 3).

## Metabolism

**FIGURE 4:**
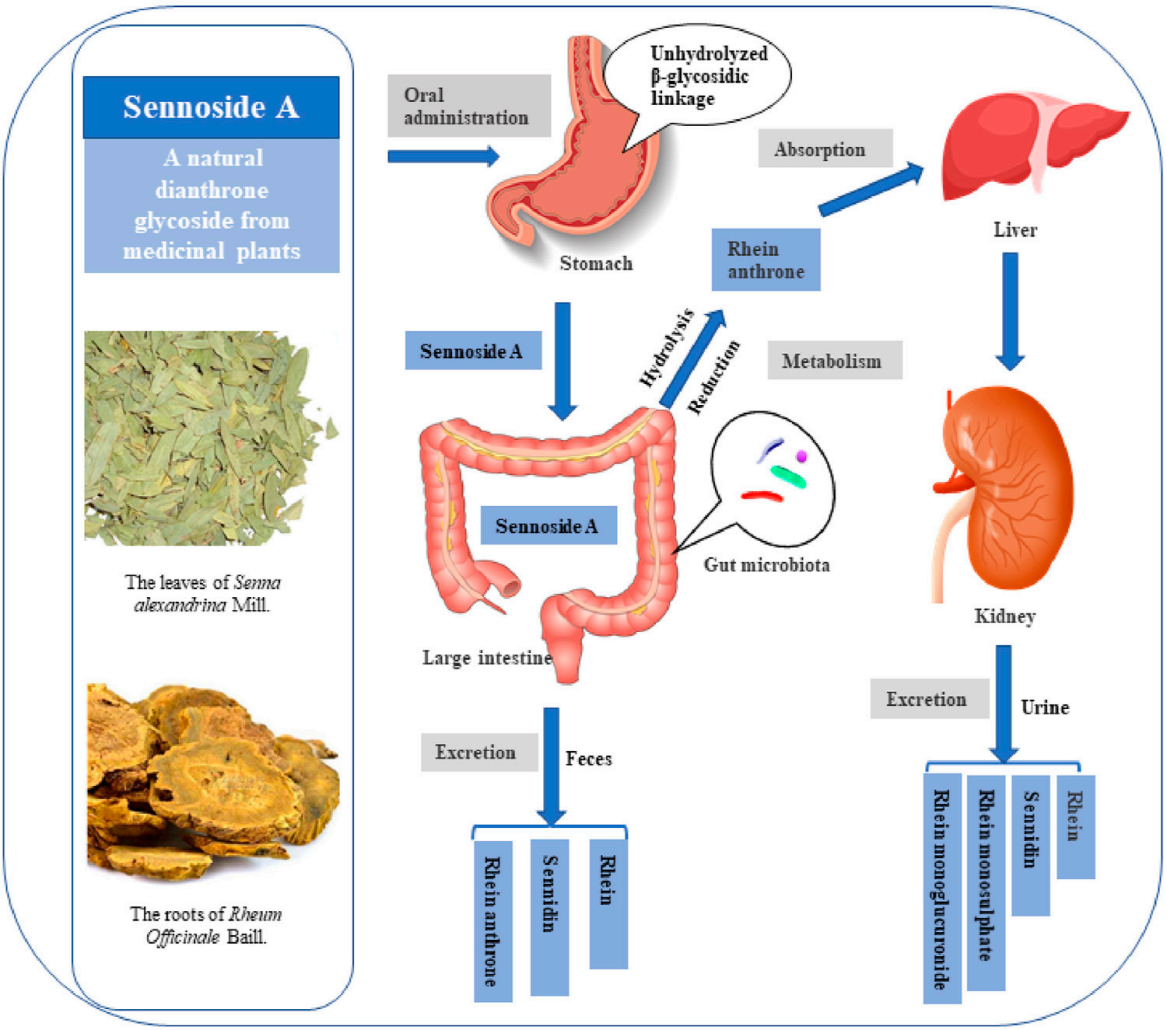
Possible metabolic pattern of sennoside A after oral administration. SA pass the stomach and small intestine without transformation and absorption. It is transformed to rhein anthrone by intestinal flora in the large intestine and therefore absorbed into enterohepatic circulation. Finally, the metabolites of SA are excreted through urine and feces.

The possible metabolic patterns of SA, including SA metabolism, absorption and excretion, were drawn as Figure 4, which illustrated the general scheme from unabsorbed glycosidic into absorbed pharmacologically active aglycon anthrone and excreted metabolites. Sennosides were not hydrolyzed by acid in the stomach nor by ß-glucosidases in the small intestine, and, therefore, were unabsorbable by intestinal epithelial cells (Dreessen et al., 1981; Lemli, 1988). After reaching the large intestine, sennosides were transformed to rhein anthrone by ß-glucosidase and reductase of intestinal microflora in two main metabolic pathways (Dreessen and Lemli, 1982; van Gorkom et al., 1999).

**FIGURE 5:**
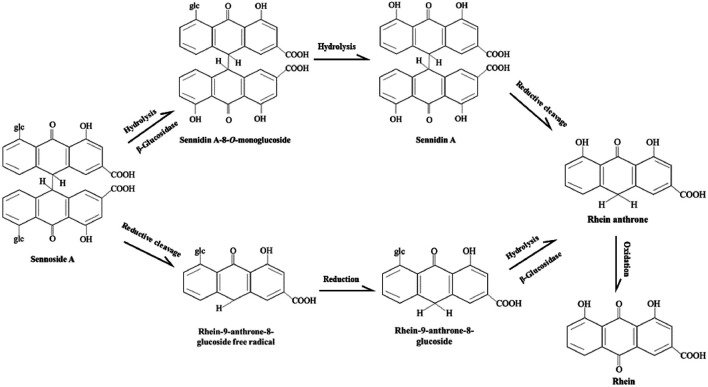
Proposed metabolism pathways of sennoside A in large intestine. The metabolic pathways of SA transforming to rhein anthrone include the hydrolysis of the O-linked sugars by bacterial β-glucosidase and the reduction of the anthrone glucoside radical by bacterial reductase. Then rhein anthrone can be transformed by oxidation into rhein.

As shown in the Figure 5, there are two proposed metabolic pathways of SA. The SA may undergo a hydrolysis reaction to generate sennidin A, then converted to rhein-anthrone by breaking the C10-10′ bond. The rhein anthrone and sennidin A were identified as SA metabolites through intestinal flora after incubation with rat and mouse feces (Kobayashi et al., 2009). In the second one, SA may first undergo hydrolysis to generate rhein-9-anthrone-8-glucoside radical, which is then converted to 8-Glucosyl-rhein-9-anthrone. Finally, the 8-Glucosyl-rhein-9-anthrone generates rhein-anthrone under the action of hydrolase. Similarly, Huang et al. found that SA was metabolized by intestinal flora into sennidin A-8-O-monoglucoside, rhein anthrone, O-methyl-hydroxy-rhein anthrone, dehydroxy-rhein anthrone, rhein (Huang et al., 2019).

Then the intestinal metabolite rhein anthrone of SA was absorbed in the colon and undergoes enterohepatic recirculation. Studies on urine and fecal excretion showed that after absorption of rhein anthrone, it was oxidized to rhein, which is excreted through urine and feces by combing with glucuronic acid or sulfuric acid. After oral administration of sennosides in the rat, rhein, sennidin, rhein monosulphate and rhein monoglucuronide were detected in the urine. The sennidin, rhein and rhein anthrone were detected in feces (Lemli and Lemmens, 1980). In addition, Yin et al. verified that 131I-SA is mainly excreted by kidney (73.5% excreted by urine and 10.5% excreted by feces). 131I-SA could quickly reach the maximum plasma concentration (Cmax, 163.316 ± 11.180 mBq/L) at 0.083 h, possessing fast blood clearance with an elimination half-life of 6.711 ± 0.564 h (Yin et al., 2017).

## Conclusion

In summary, SA has been demonstrated to be one of the valuable compounds for the prevention and therapy of constipation, obesity, diabetes, fatty liver, inflammatory and cancers. Although the pharmacological mechanism of SA has been preliminarily investigated, its molecular mechanism of pharmacological action, metabolic pathways and the toxic effects still not clear. In addition, the gastrointestinal side effects and tumorigenicity caused by long-term and high-dose use of SA remain controversial and should not be ignored. It is also uncertain whether SA itself, its metabolites or both are responsible for the pharmacological and toxicity effects. Therefore, it is necessary to further identify relevant targets and conduct clinical trials of the safety, efficacy and pharmacokinetic of SA to verify the results of SA observed *in vitro* and *in vivo*. This review focused on comprehensive research on pharmacology, toxicology, and metabolism of SA and provided a significative reference for the development and utilization of SA in the future.
